# Strategic procurement and international collaboration to improve access to medicines

**DOI:** 10.2471/BLT.16.187344

**Published:** 2017-08-23

**Authors:** Alessandra Ferrario, Tifenn Humbert, Panos Kanavos, Hanne Bak Pedersen

**Affiliations:** a LSE Health and Social Care, London School of Economics and Political Science, Houghton Street, London WC2A 2AE, England.; b Regional Office for Europe, World Health Organization, Copenhagen, Denmark.

Efficient procurement of medicines is more than just obtaining the lowest price. It is about creating a healthy market where products of good quality are available at affordable prices on a sustainable basis and at the right time.^1^ In this context, a strategic approach to procurement is vital. Such an approach should encompas all activities that might improve the efficiency of procurement – e.g. activities to minimize low-value repetitive purchases, increase the benefit of economies of scale and reduce transaction and transport costs.^2^ Here, we will provide examples of the experiences of countries in the World Health Organization’s European Region in improving the efficiency of procurement of medicines. We will also explain how international collaboration could help improve individual country’s efforts.

## Off-patent market

The efficiency of procurement of medicines that are no longer covered by patents can often be improved by switching from branded products to non-branded generic or biosimilar products.

**Fig. 1:**
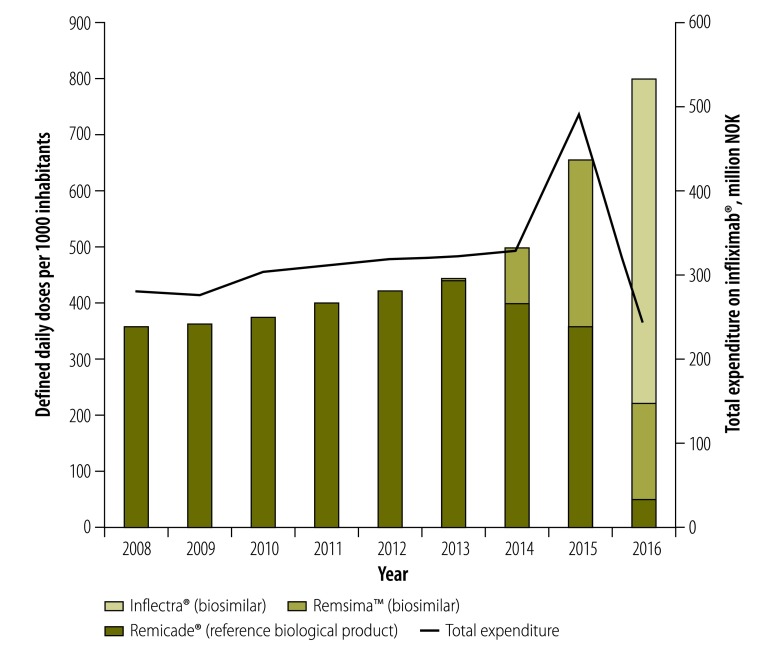
Consumption and expenditure of the reference biological product of infliximab^®^ (Remicade^®^) and two of its biosimilar products (Inflectra^®^ and Remsima™), Norway, 2008-2016

For example, although infliximab^®^ – a biological inhibitor of tumour necrosis factor-α that can be used to slow the progression of rheumatoid arthritis and other autoimmune diseases – appears superior to older treatments, the original branded product is also relatively expensive. After the first two biosimilars of infliximab^®^ received European-Union-wide marketing authorization in September 2013, Norway started switching from the original branded infliximab^®^ to the less expensive biosimilars. This switch not only allowed more patients to be treated, but also reduced overall expenditure (Fig. 1). Positive results were also achieved in Denmark, where, by the end of 2015, a biosimilar version of infliximab^®^ had almost entirely replaced the original product – and saved the Danish health service an estimated 2.6 million Euros (€).^4^ In both Denmark and Norway, initial scepticism about the effectiveness of the biosimilars – among both doctors and patients – had to be overcome by the provision of information and treatment recommendations. In Denmark, information leaflets were developed and distributed to doctors and patients and the Danish council for the use of expensive hospital medicines recommended that new and existing patients requiring infliximab^®^ be switched to a biosimilar.^4^ In Norway, annual conferences on inhibitors of tumour necrosis factor -α have been running since 2007. These conferences have offered an opportunity to familiarize the attending physicians and specialists with the biosimilar versions of infliximab^®^ and allow open discussion of any related concerns. Further reassurance came in the form of the results of a randomized study in Norway, which indicated the safety of switching patients from the original branded product, to one of its currently marketed biosimilars.^5^

NOK: Norwegian krone.

Notes: Consumption is defined as daily doses per 1000 inhabitants. A defined daily dose (DDD) is the assumed average maintenance dose for the main indication of the medicine in adults. For inflixmab it corresponds to 3.75 mg per day.^3^

Source: Based on data from Farmastat Norway.

## On-patent market

In procurement from on-patent markets, negotiation, therapeutic tendering and the use of the flexibilities offered by the agreement on trade-related aspects of intellectual property rights^6^ may all be beneficial. Therapeutic tendering is based on the ability to substitute one active ingredient with another that appears to have equivalent therapeutic effect.^7^ Medicines considered to be substitutable may be from the same therapeutic class or a different one.^7^ Within the United Kingdom of Great Britain and Northern Ireland, therapeutic tendering has been successfully implemented – e.g. in London, in the procurement of antiretroviral medicines for people living with human immunodeficiency virus (HIV).^8^ Denmark also implements therapeutic tendering. Such tendering relies on the availability of appropriate clinical guidelines –to guide the choice of medicines considered to be therapeutic equivalents. By using therapeutic tendering, the London procurement group for HIV medicines achieved annual savings of at least 10.5 million pounds sterling (£) between 2011 and 2014.^8^

Where negotiation and therapeutic tendering are impossible or inadequate to ensure access to cost–effective medicines, additional interventions may be required. In Europe, the access of many patients to useful medicines is limited by the high prices of new medicines and so-called evergreening – i.e. the extension of the life of a patent on a particular molecule or the use of alternative methods, such as the taking of secondary patents on minor variations of the same compound, to extend the effective period of market exclusivity.

The impact of high prices of new medicines on access is exemplified by the case of direct-acting antiretrovirals for the treatment of hepatitis C. Access to these medicines has been severely limited – even in high-income countries – by a lack of affordability.^9^ Issues of evergreening can limit access and cause unnecessary expenditure on older molecules. For example, in Cyprus in 2016, one month of off-patent treatment for a person with chronic myeloid leukaemia with generic imatinib cost an estimated €117.6, whereas the on-patent treatment of a patient with a gastrointestinal stromal tumour – with branded imatinib at the same dose and in the same formulation – cost an estimated €2168.4 (E Panayiotopoulou, Ministry of Health, Nicosia, Cyprus, personal communication, 2016).

Although the flexibilities offered by the agreement on trade-related aspects of intellectual property rights^6^ can be used to tackle these issues, in practice such flexibilities appear not to have been used to their full potential.^10^

## Quality of care

In Norway, the national procurement body for hospitals does not contract volume, only price. Norwegian doctors are free to decide which medicine to prescribe, but must take into consideration any relevant guidance from the procurement body. For a patient requiring treatment with an inhibitor of tumour necrosis factor-α, for example, a doctor must decide between infusion at a hospital and self-injection therapy. The doctor will take into consideration the time and cost for the patient to travel to a hospital and whether the patient is employed, would be able to inject onself and would be compliant with treatment. The price of the medicine should only be taken into account after all these other factors have been considered.

## Looking beyond lowest costs

The unit cost of a medicine should not be the only consideration when deciding which product to procure. If the use of a more expensive medicine or formulation has spillover effects that benefit the health system, it may be more cost–effective than the use of the cheapest option. In Denmark, for example, relatively expensive ready-to-use medicines were procured in large hospitals because their use freed nurses’ time and increased patient safety.^4^

## Collaboration in procurement

**Fig. 2:**
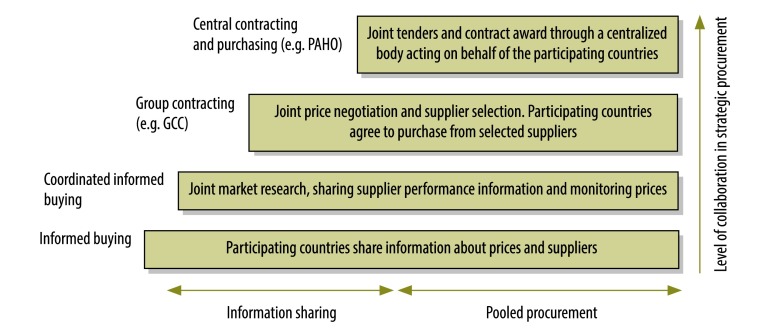
Different levels of international collaboration in the procurement of medicines

GCC: Gulf Coordination Council; PAHO: Pan American Health Organization.

Source: Based on a framework proposed by Management Sciences for Health (Medford, United States of America).

As few countries perform well in all areas of strategic procurement, international collaboration and experience sharing can be beneficial. In Europe, there are several well-established networks for the regular sharing of experiences and information on pricing, reimbursement and/or rational use of medicines. These include the network of competent authorities in pricing and reimbursement, the medicine evaluation committee, the pharmaceutical pricing and reimbursement information network and Piperska group. In addition, related collaborative initiatives also exist– e.g. in the areas of health technology assessment, horizon scanning, negotiation and procurement. The BeNeLuxA initiative, the european network for health technology assessment, the EuroScan international network, the nordic pharmaceuticals forum, the signatories to the 2016 Sophia declaration and the Visegrad group of countries are examples of such initiatives. The level of collaboration for many of these initiatives goes beyond information sharing. For example, it may include joint horizon scanning, setting standards for health technology assessment or joint negotiations or tendering for procurement. The different levels at which countries can collaborate are presented in Fig. 2.

Within the European Union, there has been joint procurement – or, at least, discussion of such procurement – of bacille Calmette–Guérin vaccine, botulinum and diphtheria antitoxins, direct-acting antiretrovirals for hepatitis C, pandemic vaccines, personal protective equipment for health workers and tuberculin. A joint procurement of vaccines by the Baltic countries is in preparation and a tendering process for the joint procurement by England and Scotland of recombinant factors VIII and IX is in place.

As yet, no international initiatives specifically address the intellectual issues associated with medicine procurement. Several possible reasons could clarify why the flexibilities offered by the agreement on trade-related aspects of intellectual property rights^6^ do not appear to have been exploited to their full extent. For example, there may be inadequate infrastructural and/or technical capacity and too much negative pressure from the pharmaceutical industry and/or other countries.^11^^,^^12^ In the many European countries where the pharmaceutical industry is an important employer and taxpayer, in addition to regulatory limitations concerning data and market exclusivity^13^, there may be fear of annoying that industry – and of any consequent retaliation. Even the smaller and less wealthy European countries that lack large pharmaceutical industries may be concerned that their full exploitation of the agreement on trade-related aspects of intellectual property rights^6^ will limit the release of new medicines within their borders. Individual country’s concerns might be reduced if they could collaborate together.

## Conclusions

The increasing calls from multiple stakeholders about the unacceptability of the high prices of new medicines require action. Some concrete initiatives have been launched and are being implemented. Improving access to medicines and patients’ outcomes is not just about prices. A process of managed entry of new medicines through horizon scanning and health technology assessment and a life-cycle approach to ensure supply security through strategic procurement are needed.^10^^,^^14^ In this context, increased collaboration between countries – in terms of sharing information relevant to health technology assessment, the managed introduction of new medicines and procurement practices – may be the first step in the right direction. However, a conducive policy environment is needed at multinational and, ideally global level. We need a revision of European Union directive 2004/48/EC – on the enforcement of intellectual property rights^15^ – that takes into account the need to provide equitable access to medicines. Such a revision, together with uptake of recommendations –made by the United Nations’ Secretary General’s high-level panel on access to medicines – on the use of the flexibilities offered by the agreement on trade-related aspects of intellectual property rights^6^ and the implementation, punishment and reporting mechanisms^12^ could provide the policy space needed to strengthen existing global, regional and national initiatives to improve access to medicines.
